# Exploring Gut Microbial Dynamics and Symbiotic Interaction in *Blattella germanica* Using Rifampicin

**DOI:** 10.3390/biology12070955

**Published:** 2023-07-03

**Authors:** Monica Cazzaniga, Rebeca Domínguez-Santos, Jesús Marín-Miret, Rosario Gil, Amparo Latorre, Carlos García-Ferris

**Affiliations:** 1 Institute for Integrative Systems Biology (I2SysBio), University of Valencia and Spanish Research Council, 46980 Paterna, Spain; mcazzaniga@ucc.ie (M.C.); rebeca.dominguez@uv.es (R.D.-S.); jesus.marin@uv.es (J.M.-M.); rosario.gil@uv.es (R.G.); 2 Genomic and Health Area, Foundation for the Promotion of Sanitary and Biomedical Research of the Valencia Region, 46020 Valencia, Spain; 3 Department of Biochemistry and Molecular Biology, University of Valencia, 46100 Burjassot, Spain

**Keywords:** *Blattella germanica*, symbiosis, *Blattabacterium*, gut microbiota, rifampicin, aposymbiont

## Abstract

**Simple Summary:**

The German cockroach *Blattella germanica* harbours two types of helpful bacteria: the obligate endosymbiont *Blattabacterium*, with a well-known function in the host metabolism, and complex gut microbiota, which are acquired mainly through faeces, whose functions still need to be fully understood. Our goal was to understand if and how these two spatially isolated symbionts communicate and interact, by disturbing them with the antibiotic rifampicin. The treatment produced deep changes in the gut microbiota, regardless of faeces addition. After their removal, most, but not all the taxa from the control population were recovered. On the other hand, it was impossible to obtain aposymbiots after treatment with rifampicin during two generations, highlighting the essential role played by the endosymbiont. Thus, quasi-aposymbiotic individuals with a reduced load of endosymbiont was obtained in the second generation and mixed with control individuals in the same environment. The microbiota were not affected by the reduction in endosymbiont. Overall, our results indicate that the gut microbiota cannot replace the essential endosymbiont and that there is no interaction between the two symbiotic systems.

**Abstract:**

*Blattella germanica* harbours two cohabiting symbiotic systems: an obligate endosymbiont, *Blattabacterium*, located inside bacteriocytes and vertically transmitted, which is key in nitrogen metabolism, and abundant and complex gut microbiota acquired horizontally (mainly by coprophagy) that must play an important role in host physiology. In this work, we use rifampicin treatment to deepen the knowledge on the relationship between the host and the two systems. First, we analysed changes in microbiota composition in response to the presence and removal of the antibiotic with and without faeces in one generation. We found that, independently of faeces supply, rifampicin-sensitive bacteria are strongly affected at four days of treatment, and most taxa recover after treatment, although some did not reach control levels. Second, we tried to generate an aposymbiotic population, but individuals that reached the second generation were severely affected and no third generation was possible. Finally, we established a mixed population with quasi-aposymbiotic and control nymphs sharing an environment in a blind experiment. The analysis of the two symbiotic systems in each individual after reaching the adult stage revealed that endosymbiont’s load does not affect the composition of the hindgut microbiota, suggesting that there is no interaction between the two symbiotic systems in *Blattella germanica*.

## 1. Introduction

Insects are the most specious group of animals, and it has been estimated that at least 15–20% of them live in symbiotic relationships with endosymbiotic bacteria that are transmitted vertically from mothers to offspring [1]. The main role of these endosymbionts is nutritional, providing the host with nutrients that are absent or scarce in their restricted and unbalanced diets [2,3]. Other insects harbour ectosymbionts, located mainly in the gut [4,5]. In fact, the mutualistic relation of these microbes with the host has proven to be important for the promotion of the insect’s health through their contribution in nutrient processing, colonisation resistance, immune system development and a variety of other traits [6].

Cockroaches (Blatellidae) are striking, as the two symbiotic systems coexist in the same individual: a primary endosymbiont, the Gram-negative *Blattabacterium cuenoti* (hereafter *Blattabacterium*) that resides in bacteriocytes, specialised cells of the fat body [7] and an extracellular microbial community, the gut microbiota, mainly present in the hindgut [8,9,10,11]. *Blattabacterium* infection was a monophyletic event that took place in the ancestor of cockroaches and termites around 300 Mya [12,13]. Over evolutionary time, *Blattabacterium* has been maintained in all cockroaches, except for the cave-dwelling *Nocticola* [14], and lost in all termites, except for the basal species *Mastotermes darwiniensis* [15]. The genome of *Blattabacterium* from many cockroach species has been sequenced and characterised (reviewed in [16,17,18,19]), unveiling its role in nitrogen metabolism, in which the urea provided by the host is degraded to ammonia by the enzyme urease encoded by *Blattabacterium*. Then, ammonia can be converted into glutamate by a *Blattabacterium*-encoded glutamate dehydrogenase or into glutamine by a host-encoded glutamine synthetase, incorporating nitrogen into the metabolism [20,21,22], as it has been corroborated by flux balance analysis and transcriptomics [21,23]. On the other hand, in the gut there exists a rich and diverse bacterial community, which plays an important role in the overall health of its host, providing defence against pathogens, modulating social behaviour by producing pheromones, supplying essential metabolites for nutrition, stimulating the cockroach’s metabolism and increasing metabolic rates [4,10,11,24,25,26]. However, it is yet unknown if there is communication between the endosymbiont *Blattabacterium* and the gut microbiota, and with the host.

The loss of *Blattabacterium* in the higher termites induced a shift from an omnivorous to a wood diet as well as the acquisition of a specialised gut microbiota [27,28], mainly located in the hindgut [28,29,30]. In termites, the gut microbes help fix nitrogen, degrading ligno-cellulose and producing essential nutrients [31]. However, it is not clear why cockroaches need their gut microbiota and whether they can somehow replace the endosymbiont essential functions. The effect of the gut microbiota on the host’s fitness was investigated in the omnivorous cockroach *Periplaneta americana* by using germ-free individuals, which showed severe growth and development defects and a starvation-like transcriptional response [32,33]. However, in our laboratory, germ-free *B. germanica* does not show any alteration in the development of the insect but shows reduced urate deposits in adults eliciting, as in *P. americana*, a starvation-like response (unpublished results).

Regarding the acquisition of the two symbiotic systems from one generation to the next one, in *B. germanica* only *Blattabacterium* was present in the embryos, confirming that it is vertically transmitted to the offspring [8]. Thus, at hatching, the hindgut is sterile, and the gut bacterial load increases about two orders of magnitude from the first to the second nymphal stage, indicating that the establishment of the gut microbiota happens early in development [8,34]. In this cockroach species, the high similarity of bacterial composition in the faeces and gut suggested that coprophagy plays an important role in the transmission, re-colonisation and succession of the gut microbiota [25,35,36,37,38]. Lately, the treatment with antibiotics demonstrated that the acquisition and establishment of the bacterial communities occurs by horizontal transfer mainly via faecal contents, since the recolonisation after antibiotic treatment in the second generation is faster when faeces are added to the diet [34,35,38]. In this sense, antibiotic therapy is a powerful tool that can be used to selectively affect these two symbiotic systems, providing a unique opportunity to study whether the gut microbiota somehow complements *Blattabacterium* functions, which may imply a dialogue between them and the host [38]. It is worth mentioning that, even though antibiotic treatment could have negative effects on the host physiology, it is the most widely adopted method to eliminate endosymbionts. Alternative methods such as lysozyme and heat treatments on cockroaches had a similar deleterious effect on host physiology than those observed when treated with high doses of antibiotics [39]. The effect of antibiotics on the host has been widely studied in aphids, and the main conclusion is that the negative effects associated with aposymbionts treated with antibiotics were not attributable to a direct effect of the antibiotic [40].

Rifampicin is widely recognised as one of the most effective and broad-spectrum bactericidal antibiotics. In previous works, we demonstrated that *Blattabacterium* is susceptible to rifampicin in adults only during its extracellular phase in the ovaries, when it leaves the protection of the bacteriocytes to infect the mature oocytes. For this reason, the reduction in the endosymbiont population is not observable in the treated generation, but in its progeny [38]. The taxonomical composition of the gut microbiota is also altered by rifampicin, showing a reduced diversity after 10 days of treatment [38]. Cockroaches have also been treated with doxycycline in one generation and the *Blattabacterium* load was no detected by PCR in the offspring [41]. Other antibiotics such as vancomycin, ampicillin and kanamycin also affect the gut microbiota but not the endosymbiont [34,35,42,43,44], while rifampicin is one of the tested antibiotics that can be used to disturb both symbiotic systems. Therefore, in order to empirically determine the role of the gut microbiota in host fitness and if it can somehow supply the endosymbiont function, it may be necessary to generate a stable cockroach population with a null load of *Blattabacterium,* while preserving a healthy gut microbiota. This would allow us to address the question of whether there is interaction between both symbiotic systems and with the host. A previous experiment, to reduce as much as possible the presence of the endosymbiont in *B. germanica* using rifampicin, failed to obtain a homogeneous and persistent aposymbiotic population [45]. To affect the gut microbiota as little as possible, rifampicin with faeces from a control population were supplied to the adult for a short period of time, allowing for the posterior recovery of the microbiota after treatment cessation. In the present work, we carried out an experiment to examine specifically the effect of the rifampicin treatment on the gut microbiota and their recovery in one generation. Next, we address the generation of an aposymbiotic *B. germanica* population by extending the treatment during the whole adult stage over several consecutive generations, studying the developmental and reproductive effects of the antibiotic. Finally, we use rifampicin to generate, in the second generation, a stable quasi-aposymbiotic population of *B. germanica* with a much-reduced load of *Blattabacterium* to investigate whether the composition and structure of the microbiota are affected by a reduced load of *Blattabacterium* and the possible interaction between the two symbiotic systems.

## 2. Materials and Methods

### 2.1. Blattella germanica Maintenance and Dissection

*B. germanica* was reared in climatic chambers (Inkoa Ca00/15, Bizkaia, Spain) at the Institute for Integrative Systems Biology (I2SysBio, University of Valencia-CSIC) at 25 °C, 60% humidity and a photoperiod of 12 h light/12 h darkness (cool white fluorescent tubes, irradiance around 20 W/m^2^ on the containers). Insects were fed dog pellets (Teklad global 21% protein dog diet 2021C, Envigo) and water ad libitum was supplied. When required, rifampicin (Alfa Aesar, Kandel, Germany) was provided with the water at 0.1 or 0.2 mg/mL, depending on the experiment. Faeces from control population were added to the diet when needed.

At selected time points (see next section), three adult females from each experiment were anesthetised under a steam of CO_2_, and fat body and/or hindgut were dissected under a Stereo Microscope SZ61 (Olympus, Barcelona, Spain). The tissues were collected using Krebs-Ringer bicarbonate buffer (Sigma-Aldrich, St. Louis, MO, USA), frozen in liquid nitrogen and stored at −80 °C until use. Only females were selected for dissection, and each time a female was dissected, a male individual was removed from the population to avoid sex-bias effects.

### 2.2. Experimental Design

**Figure 1:**
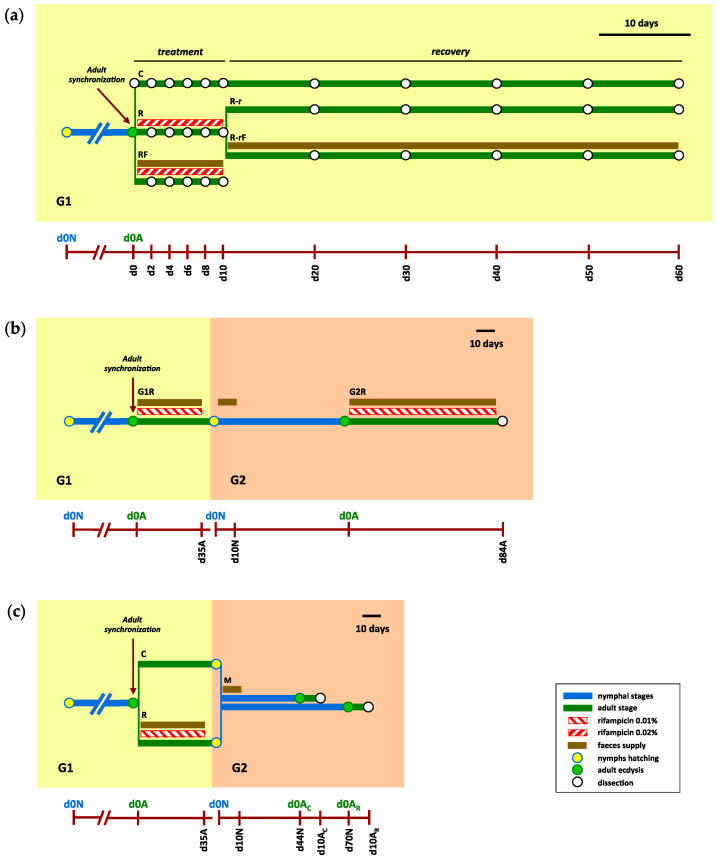
Overview of the three experimental designs. (**a**) Rifampicin treatment and recovery during a single generation. The experiment was divided in two phases: treatment, in which rifampicin was administered at 0.2 mg/mL for 10 days, and recovery, after cessation of the rifampicin treatment. A nymphal population was created (0dN) and an adult synchronised population was generated (d0A) selecting individuals that passed to adult stage in a period of 48 h. Three different populations were generated at d0: control (C), treated with rifampicin (R) and treated with rifampicin and faeces (RF). Samples were collected during the treatment phase every 2 days (d2 to d10). After 10 days, the antibiotic treatment was ceased, starting the recovery (r) phase. The R population was divided into two sub-populations: R-r and R-rF, without or with faeces added during the recovery, respectively. Samples were collected during recovery phase every 10 days (d20 to d60). At each sampling time indicated in the timeline, the dissection of three female cockroaches was carried out and hindguts were collected. (**b**) Obtaining an aposymbiotic population. The experiment expands for two consecutive generations, G1 and G2. At G1, a synchronised sex-balanced adult population (d0A) was obtained as in (**a**). This population was treated with rifampicin at 0.1 mg/mL and supplied with faeces (G1R population). After 35 days (d35A at G1), rifampicin treatment was ceased to allow for nymphs to hatch (0dN at G2) in an antibiotic-free environment. Faeces from a control population were supplied for the first ten days (until d10N and G2). The adults obtained from this nymphal G2 population were treated again with 0.1 mg/mL rifampicin during the adult period with faeces supplied (G2R population). The last female adults were collected and dissected after 84 days (d84A). (**c**) Effect of *Blattabacterium* load on the structure and composition of the gut microbiota. The experiment expands for two consecutive generations, G1 and G2. At G1, a synchronised sex-balanced adult population (d0A) was obtained as in (**a**) and divided into two populations: control (C) and treated with rifampicin at 0.1 mg/mL and supplied with faeces from a control population (R) during 35 days (up to d35A at G1) to allow for hatching in an antibiotic-free environment, as in (**b**). Once nymphs hatched in both populations, they were separated from the adult individuals and joined together to form a unique mixed population (M), where faeces were added for the first 10 days (up to d10N at G2). Then, each adult female that appeared in the M population was separated individually, and after 10 days dissected to collect the fat body and the hindgut of the same individual. Adult ecdysis occurred in two waves, one corresponding to adults from the control population in G1 (d0A_C_, that were dissected at d10 A_C_), and other corresponding to adults from the rifampicin-treated population in G1 (d0A_R_, dissected at d10 A_R_) that showed developmental delay.

An overall presentation of the experiments followed in this work is illustrated in Figure 1.

#### 2.2.1. Effect of Rifampicin Treatment and Microbiota Recovery during a Single Generation

Adult cockroaches were collected to start a sex-balanced synchronised population between 0 and 48 h after adult ecdysis (Figure 1a). Before any treatment, three females were dissected (d0), and their hindguts were collected. In the treatment phase, three populations were established: control (C, founded by 46 females and 46 males), treated with rifampicin (R, founded by 71 females and 71 males) and treated with rifampicin and supplied with faeces from a laboratory control population (RF, founded by 25 females and 25 males). Rifampicin was administered at 0.2 mg/mL for 10 days, and every two days (d2 to d10), three females of each population were dissected and their hindguts were collected. We wanted to know how rifampicin treatment affected the microbiota and whether the effect could be attenuated by supplying faeces with the diet during treatment. Then, after the 10 days of treatment, no more antibiotic was provided and the recovery (r) phase was initiated. Then, the R population was divided in two sub-populations, without or with faeces added (R-r, founded by 28 females and 28 males, and R-rF, founded by 27 females and 27 males). These sub-populations and the C population were maintained for 50 more days (until d60), and three females from each population were dissected every 10 days (d20 to d60) to analyse their hindguts. In the two treated sub-populations, we wanted to investigate how the gut microbiota were recovered once the antibiotic was removed and whether faeces helped in the recovering process. In summary, 31 time points and 93 hindgut samples were studied: (i) 48 from the treatment phase of which 18 come from the control population (3 samples correspond to Cd0 and 15 to C2 to C10), 15 from the R population and 15 from the RF population; and (ii) 45 from the recovery phase (corresponding to d20 to d20), 15 from the C population, 15 from the R-r population and 15 from the R-rF population.

#### 2.2.2. Obtaining an Aposymbiotic Population

The experiment started from a synchronised adult population (d0A at the first generation, G1, founded by 48 females and 35 males) (Figure 1b). Rifampicin was provided during the adult stage at 0.1 mg/mL to lessen its effect on *Blattabacterium* and the microbiota, and faeces were added to reduce the impact of rifampicin treatment on the gut microbiota (G1R population). After 35 days (d35A), the antibiotic was removed and around 250 newly hatched nymphs were selected during a period of seven days to start the second generation (d0N at G2) and faeces were added for 10 days (d10N) to make sure they were able to acquire the control gut microbiota. When G2 cockroaches started to reach the adult stage, they were collected every 48 h (considering d0A at G2 the first ecdysis). Rifampicin was supplied during all the adult stage at 0.1 mg/mL in order to reduce even more the *Blattabacterium* load in the next generation, and faeces were also added (G2R population). Finally, after 84 days (d84A), six adult females were dissected and their fat bodies were collected to quantify *Blattabacterium*.

#### 2.2.3. Effect of *Blattabacterium* Load on the Structure and Composition of the Gut Microbiota

A synchronised adult population was initiated as described above and divided into two subpopulations (Figure 1c): control (C, founded by 20 females and 15 males), and treated with rifampicin at 0.1 mg/mL and supplied with faeces from a control population (R, founded by 48 females and 35 males) during 35 days (d35A). Once the nymphs hatched, a mixed population (M) was established by joining 70 newly hatched nymphs from the C population and 140 newly hatched nymphs from the R population, and faeces from the control population were supplied as input for microbiota implantation in the new population for 10 days. The mixed population shared the same habitat until the individuals reached the adult stage, which occurred in two waves; one that began at around 44 days (d44N) and the other at around 70 days (d70N), coming from the C and R population, respectively. At this moment, the females were individualised (d0A_C_ and d0A_R_). On day 10, female adult cockroaches were dissected and their fat body and hindgut were collected to quantify the *Blattabacterium* population and to analyse the composition of the gut microbiota, respectively. Altogether, 77 individuals (male and female) passed to the adult stage and the hindgut and fat body samples of the 38 females were collected and analysed.

### 2.3. DNA Extraction, Sequencing and Quantitative PCR (qPCR)

Hindgut total DNA was extracted following the protocol of Jet Flex Genomic DNA Extraction kit (Genomed, Löhne, Germany) with slight modifications, such as lysozyme treatment (20 mg/mL, 4 h at 58 °C) [21]. The obtained DNA was used for 16S rRNA metabacording and metagenomic studies. Sequencing was conducted at the Sequencing and Bioinformatics Service facilities of FISABIO (Foundation for the Promotion of the Health and Biomedical Research of Valencia Region, Valencia, Spain). In samples intended for metabarcoding analysis, total DNA was used as template for the amplification of the V3–V4 region of the bacterial 16S rRNA gene, and the amplicons were sequenced following the recommended protocol 16S Metagenomic Sequencing Library Preparation for the Illumina Miseq System and using the Nextera XT Index Kit v2. For metagenomic studies, sequencing was carried out using Nextera XT DNA Library Preparation Kit, following Illumina recommendations for NextSeq 500 (2 × 150 bp).

The DNA extraction from fat body was carried out following the method described in [35]. DNA was quantified with Qubit 2.0 Fluorometer (Thermo Fisher, Waltham, MA, USA) and used for qPCR analyses. The absolute number of copies of *Blattabacterium* genome per nanogram of total fat body DNA was determined by qPCR using the *ure*C gene (accession number NC_013454.1), whereas the eukaryotic gene *actin*5C (accession number AJ861721.1) was used as internal control because its quantification per nanogram of DNA must remain constant since the majority of DNA extracted from the fat body is eukaryotic. Specific primer pairs were used for amplifying *ure*C and *actin*5C gene fragments as previously described [38]. Purified PCR-amplified products from *ure*C and *actin*5C genes were serially diluted 10-fold to generate a standard curve for each gene. qPCR reactions were carried out on an ArialMx Real-Time PCR System (Agilent Technologies, Böblingen, Germany), using SYBR Green as fluorescent reporter. Cycling conditions and fluorescent measurements were performed as previously described [38]. All reactions were run in triplicate. Wilcoxon test (adjusted *p*-value by FDR method) was applied to analyse statistical differences.

### 2.4. Hindgut 16S rRNA Metabarcoding and Metagenomic Analysis

The taxonomical composition of the hindgut microbiota was determined after the 16S rRNA sequencing data with QIIME 2 version 2018.8 (https://qiime2.org/, accessed on 13 August 2018). Quality filtration, denoising and merging of paired-end reads were performed using the DADA2 tool [46] in QIIME 2 environment. The end product of DADA2 is an amplicon sequence variant (ASV) table, a higher-resolution analogue of the traditional OTU table, which records the number of times that each exact amplicon sequence variant was observed in each sample.

For metagenomics data analysis, the quality control checks on raw sequence data were conducted with FastQCv0.11.6 [47]. The sequences were then trimmed with PRINSEQ [48] and the fragments were joined with Fastq-Join. Taxonomic assignments to achieve high accuracy and fast classification speeds was carried out with Kaiju software [49]. Finally, the specific taxonomic levels were assigned with addTaxonNames, implemented in Kaiju software. Then, the PCoA was carried out using phyloseq v1.30.0 [50] and vegan v2.5-6 [51] packages implemented in the R software, taking into account the data compositionality and sparsity, by performing a centred log-ratio transformation to the obtained data [52,53].

### 2.5. Biodiversity Analysis

Alpha diversity was calculated through the Shannon index [54] and a boxplot was made to graphically represent the differences in species’ diversity between samples. Beta diversity analysis was performed by exploring the composition through principal coordinate analysis (PCoA) based on Bray–Curtis distances that produces a set of uncorrelated (orthogonal) axes to summarise the variability in the dataset, expressed with an eigenvalue. The obtained data were statistically tested with the Wilcoxon signed-rank test implemented in the vegan R package [51]. Differential abundance analysis of ASVs was performed using LEfSe (Linear discriminant analysis Effect Size), a tool that is hosted on a Galaxy web application (https://huttenhower.sph.harvard.edu/galaxy/, accessed on 1 May 2019). The taxa that had LDA score higher than 2 and *p*-value < 0.05 were considered. Then, in order to analyse the microbial community structure and the changes in the presence of identified taxa over the time, heatmaps were performed using gplots package implemented in the R software. In this case, in order to better visualise the relative abundance of each taxonomic group, the average of the three replicas of each condition was displayed on the heatmap.

## 3. Results and Discussion

### 3.1. Effect of Rifampicin Treatment and Subsequent Microbiota Recovery during a Single Generation

In order to design an effective treatment with rifampicin to eliminate or reduce the endosymbiont, trying to disturb the gut microbiota as little as possible, we need (i) to understand how this antibiotic affects the microbiota during the treatment, (ii) how the microbiota recover once the antibiotic is removed and (iii) if it is possible to minimise the negative effect and speed up the gut microbiota recovery by adding faeces to the diet. To do this, we carried out the analysis of 16S rRNA gene (region V3–V4) during the treatment phase with rifampicin, and in the recovery phase after treatment cessation in the same generation (Figure 1a). Three females were collected before any treatment (d0). During the treatment phase, 45 females were collected every two days at five time points in three different populations (C, R and RF). During the recovery phase, 45 females were collected every ten days at five time points in two sub-populations derived from the R population, without or with faeces added (R-r and R-rF) plus a control (C) non-treated population. It is worth mentioning that, in a previous experiment, we treated one generation with rifampicin without recovery and the effect was seen in the next generation [38].

The total number of reads obtained from the sequencing after the quality check amounted to 6,176,538 with an average number of 68,628 sequences per sample (maximum 1,256,329; minimum 26,740). The gut microbiota composition was analysed at the family level (or the lowest possible taxonomic level). We detected 62 different families (or higher taxonomic levels) distributed in 14 different phyla in all samples considered (Tables S1 and S2). The taxa detected in the control population were in agreement with those found in previous studies in our group [35,38].

**Figure 2:**
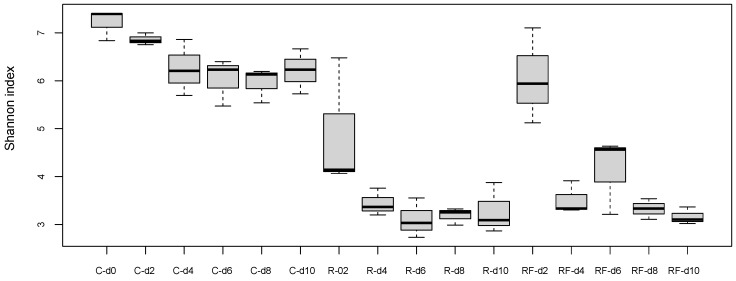
Alpha diversity analysis (Shannon index) at the treatment phase. Three populations were analysed: control sample (C), treated with rifampicin (R) and treated with rifampicin and faeces (RF). Samples were collected in five time points as indicated in Figure 1a (d2 to d10). An additional sample was taken at d0 before the split in the three populations. *p*-values corresponding to the statistical analysis for all the pairwise comparison are available in Table S3.

**Figure 3:**
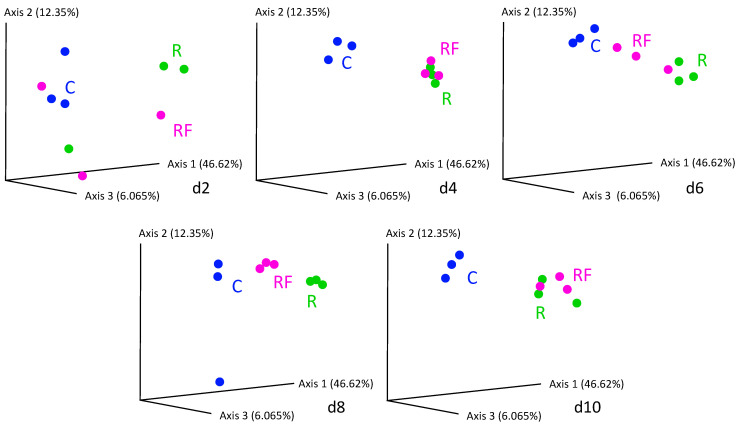
PCoA analysis based on Bray–Curtis distances corresponding to the treatment phase of the samples d2 to d10. Blue dots correspond to control population (C) samples. Green dots correspond to the rifampicin-treated population (R) and pink dots correspond to the rifampicin-treated population supplied with faeces from a control population (RF). See Figure 1 for details.

At the treatment phase, the comparative analysis of bacterial communities at the five points of the control and rifampicin-treated samples (d2 to d10) revealed drastic changes in the alpha-diversity of the gut microbiota in the treated populations (R and RF), mainly after 4 days of treatment and remaining stable from d6 to d10, as reflected by the Shannon index values (Figure 2). Except at d0, control samples can be grouped (named C) because no significant differences were found with Adonis test (*p*-value = 0.014 for C-d0 to C-d10, *p*-value = 0.061 for C-d2 to C-d10). When the faeces were supplied, a tendency to delay the effect of rifampicin was apparent after two days of the antibiotic treatment (d2). However, no significant differences were detected in the comparisons between C and R-d2 samples (*p*-value = 0.1917) or between C and RF-d2 samples (*p*-value = 0.7676). The rest of the comparisons between C and the two treated populations (sampling times d4 to d10) showed significant differences (Table S3), indicating that there is a drastic effect on the taxa sensitive to rifampicin, which is complete at four days of treatment. The supply of faeces could slightly delay the effect of rifampicin in reducing microbial diversity at d2, whereas non-significant differences were found between the treated populations R and RF at any sampling time (Table S3). These results indicate that the same level of diversity is reduced regardless of whether faeces are supplied or not during the treatment and that faeces are not able to compensate for the drastic effect of the antibiotic. In the previous work, a drastic effect was postulated at 10 days of treatment [38], and now we have found that the effect is even faster as it occurs at four days. For the beta-diversity analysis, we performed a PCoA based on Bray–Curtis distances (Figure 3). R and RF populations appear separated from the C population samples except for day 2, and they grouped together mainly at the end of the treatment (d10), which is consistent with the alpha-diversity results.

We carried out a heatmap (Figure S1) based on the relative abundance of the families in each experimental condition (Table S1) at three sampling times: d2 and d4, and d10 for studying the initial and final effect of rifampicin over the bacterial communities, respectively. C and RF-d2 samples clustered together and were separated from the other sampling-time groups, indicating different taxa composition. These results agree with the Shannon and PCoA analyses (Figure 2 and Figure 3) and confirm that adding faeces from the beginning could delay the rifampicin effect on the gut microbiota.

**Figure 4:**
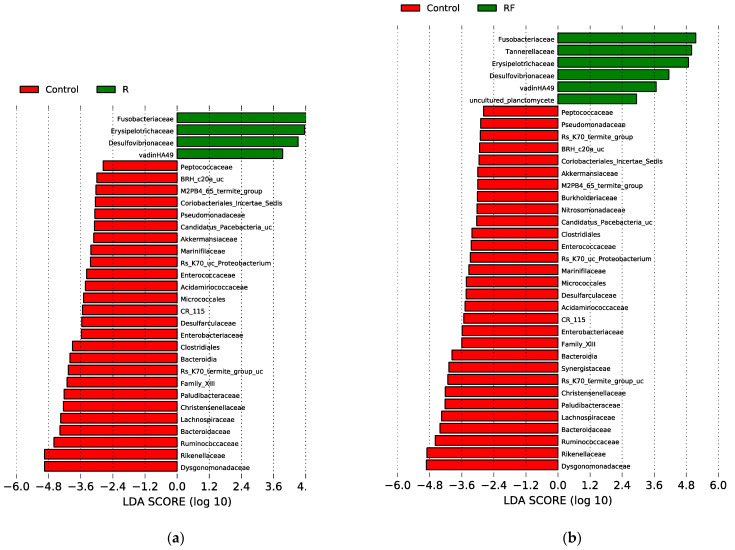
LEfSe graphs comparing C, R and RF samples at 10 days of treatment (d10). (**a**) C vs. R. positive LDA scores are assigned to the overrepresented taxa in R (green) and negative LDA scores to taxa overrepresented in C (red). (**b**) C vs. RF. positive LDA scores are assigned to the overrepresented taxa in RF (green) and negative LDA scores to taxa overrepresented in C (red). Only taxa with LDA scores higher than 2 and *p*-values lower than 0.05 were consider as significant and shown.

We have identified both the rifampicin-resistant and sensitive bacterial families in all samples in antibiotic-treated populations (R and RF) at d10. To identify taxa with statistically significant different abundance between groups of samples, we carried out the linear discriminant (LDA) effect size (LEfSe) analysis for the last sampling time (d10) in all three populations (LDA score higher than 2 and *p*-value < 0.05) (Figure 4) and detected significant differences corresponding to C-d10 vs. R-d10 (a), and C-d10 vs. RF-d10 (b). Regarding resistant taxa, four families were significantly more abundant in the R population (Fusobacteriaceae, Erysipelotrichaceae, Desulfovibrionaceae and vadinHA49) and six families in the RF population (the previous ones plus Tannerellaceae and uncultured planctomycete) compared to the C population. These taxa, except for vadinHA49, were also detected as rifampicin-resistant by Rosas and collaborators [38]. As for the rifampicin-sensitive ones, 30 and 26 taxa where more abundant in the C population, compared with RF and R, respectively. The most sensitive, according to the LDA score, were Dysgonomonadaceae, Rikenellaceae, Ruminococcaceae, Bacteroidaceae, Lachnospiraceae, Christensenellaceae, Paludibacteraceae, Rs-K70 termite group_uc, Family XIII and Bacteroidia (Figure 4 and Table S1). Finally, no family with significant differences was detected in the LEfSe analysis of the R-d10 vs. RF-d10 comparison. These results were consistent with what was found in the heatmap as well as with previous results [38].

**Figure 5:**
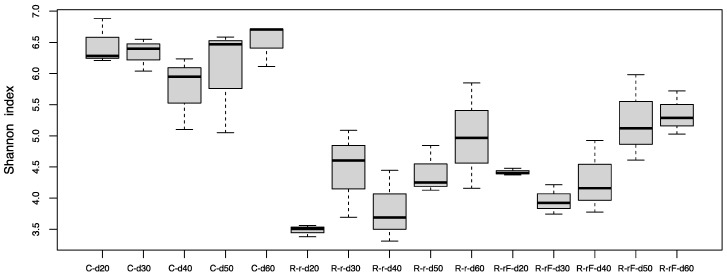
Alpha diversity analysis (Shannon index) at the recovery phase. Three populations were analysed: control (C) and recovered after treatment cessation without (R-r) and with faeces from a control population (R-rF). Samples were collected in five time points as indicated in Figure 1a (d20 to d60). *p*-values corresponding to the statistical analysis for all the pairwise comparison are available in Table S3.

To study the recovery of the gut microbiota after cessation of the antibiotic treatment, female cockroaches were collected every ten days at five different time points (d20 to d60, spanning the 50 days of the recovery phase) in the C, R-r and R-rF, populations (Figure 1a). Control samples during recovery did not show significant differences with the Adonis test (*p*-value = 0.055 for C-d20 to C-d60), being grouped for statistical analyses. We found significant differences in alpha-diversity (Shannon index) between the C group and R-r and R-rF samples during all the recovery phase (Table S3). However, non-significant differences were found between the R-r and R-rF populations at any sampling time (Table S3). These results indicate that, even after 50 days of recovery (d60), the structure of the gut microbiota is different from the control population. In addition, faeces do not speed microbiota recovery as both populations reached a similar microbiota composition after rifampicin treatment cessation (Figure 5).

**Figure 6:**
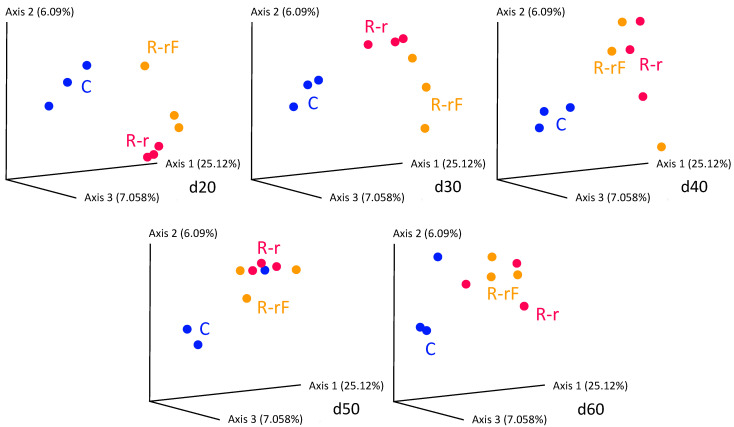
PCoA analysis based on Bray–Curtis distances corresponding to the recovery phase of the samples d20 to d60. Blue dots correspond to control population (C) samples. Red dots correspond to the rifampicin-treated population recovered without faeces (R-r) and orange dots correspond to the rifampicin-treated population recovered with added faeces (R-rF). See Figure 1 for details.

However, in the beta diversity analysis (Figure 6), C samples were bundled together, and the recovery populations R-r and R-rF clustered in a separated region. This result indicates that, although there was some recovery at d60, it was incomplete in terms of species richness. Similar results had been obtained for the gut microbiota in follow-up studies of humans treated with antibiotics and after treatment cessation [55].

Similar to the treatment phase, a heatmap based on the relative abundance of the families in each experimental condition was carried out (Table S2) at sampling time d20 for analysing the first recovered taxa, and d50 and d60 for studying the reestablishment of the bacterial communities in the presence/absence of faeces (Figure S2). The C and R-rF-d60 samples appear more closely related with respect to all other conditions, including R-r-d60, despite the non-significant differences between R-r and R-rF samples at d60 in alpha-diversity (Table S3). As expected, the R-r-d20 samples were the most distant among all the others.

**Figure 7:**
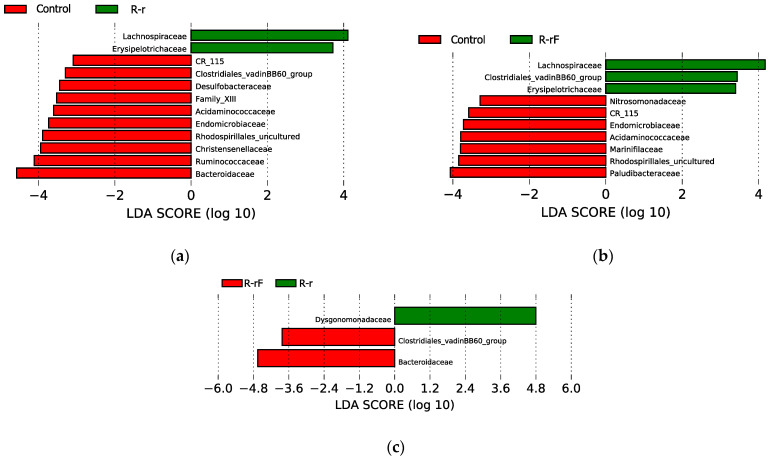
LEfSe graphs comparing C, R-r and R-rF samples at 50 days of treatment (d60). (**a**) C vs. R-r. Positive LDA scores are assigned to the overrepresented taxa in R-r (green) and negative LDA scores to taxa overrepresented in C (red). (**b**) C vs. R-rF. positive LDA scores are assigned to the overrepresented taxa in R-rF (green) and negative LDA scores to taxa overrepresented in C (red). (**c**) R-r vs. R-rF. positive LDA scores are assigned to the overrepresented taxa in R-r (green) and negative LDA scores to taxa overrepresented in R-rF (red). Only taxa with LDA scores higher than 2 and *p*-values lower than 0.05 were consider as significant and shown.

We also carried out a LEfSe analysis to identify taxa with statistically significant different abundance between groups of samples for the last sampling time of the recovery phase (d60) in all three comparisons (Figure 7): C-d60 vs. R-r-d60 (a); C-d60 vs. R-rF-d60 (b) and R-r-d60 vs. R-rF-d60 (c). Compared to the C population, only two families (Lachnospiraceae and Erysipelotrichaceae) were significantly more abundant in the R-r population and three in the R-rF one (the previous two and Clostridiales vadinBB60 group). On the other hand, only ten and seven families where more abundant in C compared with R-r and R-rF, respectively, corresponding to the rifampicin-sensitive taxa that have not been recovered to control levels, without or with faeces supplied. Three families (CR_115, Endomicrobiaceae and Acidaminococcaceae) are not recovered in both rifampicin-treated populations, regardless the addition of faeces or not.

In the LEfSe analysis comparison between R-rF-d60 and R-r-d60, the significant differences between the recovery with or without faeces only affect three families. The Bacteriodaceae and Clostridiales vadinBB60 groups were more abundant in the R-rF sample, suggesting that the faeces treatment was essential in the recovery of these two important taxa in the *B. germanica* microbiota, while Dysgonomonadaceae was more abundant in the R-r-d60 sample. However, this taxon is one of the most abundant in all samples, and over time it becomes more abundant in R-r than in R-rF (Table S2). These results indicate that, although most taxa that were sensitive in the treatment are recovered at d60, not all are recovered to control levels, and the recovery populations R-r and R-rF do not recover equally although they are more affected without faeces.

The partial reestablishment of taxa in the hindgut microbiota of adult females of *B. germanica* with or without supply of faeces indicates that not only faeces, but other factors such as environment and diet, must also be involved in shaping the bacterial community once it has been affected by the rifampicin treatment, as previously postulated [38]. For example, the order of colonisation of the sterile hindgut at birth must be important, because in our experiment the recolonisation initiates over an adult gut with microbiota already present. Furthermore, the fact that some lost or greatly reduced taxa can be recovered without adding faeces in the R-r population indicates that traces of these bacteria must be present in the faeces or in the gut at a very low abundance while the antibiotic pressure is maintained, and they were able to re-colonise the gut niche once it was removed [38], although at a slower rate than when faeces were added.

### 3.2. Obtaining an Aposymbiont Population

In the first experiment, we studied, in one single generation, the effect on the gut microbiota of treatment with rifampicin for 10 days, and their recovery after treatment cessation. In order to assess whether there exists a communication between the microbiota and *Blattabacterium,* we designed a second experiment to obtain an aposymbiotic population by cumulative treatment with rifampicin at low dose during the adult stage over several generations (Figure 1b).

**Figure 8:**
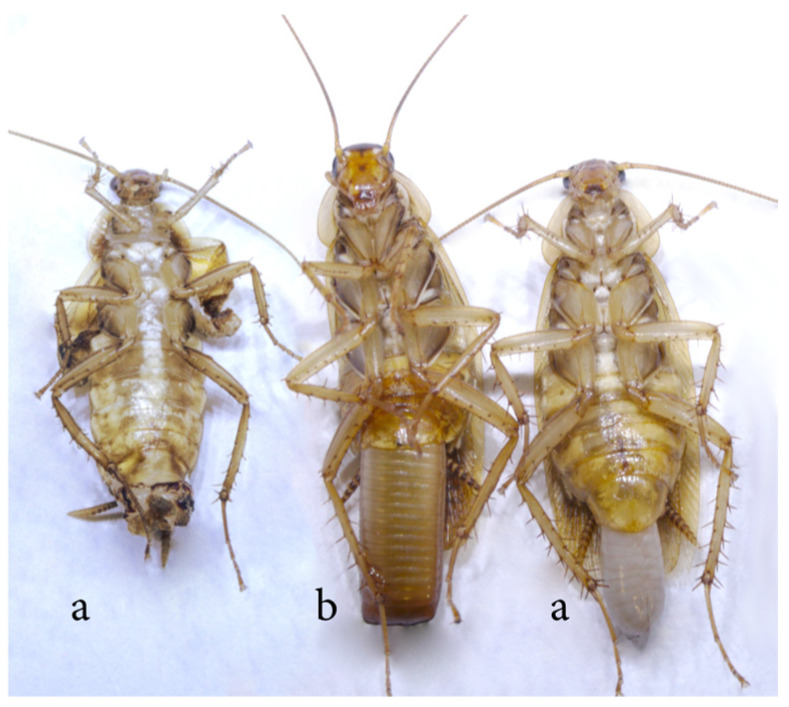
Morphological differences between two quasi-aposymbiotic adult females from the rifampicin-treated population G2R (a) and a female from a laboratory control population (b).

In a previous experiment, we tried to generate an aposymbiotic population using rifampicin over generations during the first 12 days of the adult stage. However, the window treatment was too short due to the developmental delay of quasi-aposymbiont individuals in the second generation, allowing them to escape the effect of the rifampicin and recovering the normal load of *Blattabacterium* in the third generation [45]. To avoid that, in this improved experimental design, a G1 synchronised adult population was treated with rifampicin 0.1 mg/mL (G1R) during 35 days, until oothecae maturation. To minimise the effect of the antibiotic, faeces were added throughout the treatment. Newly hatched nymphs were separated from the adults to generate the next generation (G2) and faeces from a control population were added for 10 days to guarantee the correct microbiota acquisition. In previous studies, the delay in the development, as a consequence of the *Blattabacterium* load reduction, was already evident in the time required to reach the adult compared with non-treated individuals [45]. In the present experiment, adult ecdysis started at around 70 days of the nymphal period, whereas in the control individuals it was at around 45 days. Every 48 h, new adults were collected from the population and rifampicin treatment and faeces were added (G2R). Only 28 individuals reached the adult stage, nine females and nineteen males, but three females and five males died soon after, leaving only six females to start the third generation (G3). Morphological differences can be observed between females originated from a laboratory control population and those six derived from the treated population (see Figure 8 as an example of two of them). The latter presented pale colour of the cuticle, amorphous wing development and abortive oothecae (a), compared to the control one (b). Thus, due to these abnormal reproduction and development aspects, G3 was never reached. Even though, as stated in the introduction, the possible impact on host physiology of the antibiotic treatment cannot be ruled out, we consider that the best explanation for the changes observed in the two analysed fitness parameters is that *Blattabacterium* is essential for host physiology. A similar result was obtained when *B. germanica* was treated with aureomycin to reduce the endosymbiont load. At G2, the nymphs had a high mortality, and those that did not die grew slowly, their development was prolonged and there were problems with cuticle synthesis and reproduction [39]. Moreover, alternative non-antibiotic treatments to eliminate the endosymbionts produce similar phenotypes in the progeny.

**Figure 9:**
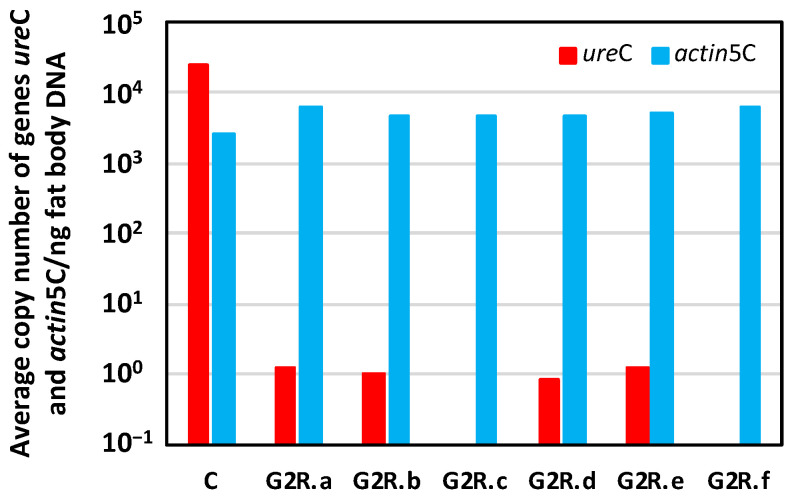
qPCR of six quasi-aposymbiotic females to determine the *Blattabacterium* load in the fat body. Log representation of the average copy number of genes *ure*C (red) and *actin*5C (blue) per nanogram of fat body DNA for each adult female dissected at d84A of G2R. G2R.a to G2R.f correspond to each of the six females that reached the end of the experiment and were dissected. C corresponds to a control adult female.

After 84 days of the adult period G2R, as G3 had not been reached (the normal period is around 45 days, as stated above), we decided to dissect those six surviving females and to estimate the *Blattabacterium* load to verify whether our prediction was achieved (females are indicated as G2R.a-f in Figure 9). The *Blattabacterium* load, as determined by the qPCR analysis of the amount of *ure*C, varied significantly, about five orders of magnitude, between the G2 control (C) and the G2R adult females, two of them (G2R.c and G2R.f) being under the detection limit of the qPCR. The number of copies of the *actin*5C gene, as markers of host genome copies, was stable in both population samples.

We concluded that, when the rifampicin treatment was extended during the whole adult period of G1, the *Blattabacterium* load was reduced in all the individuals of G2 (we call them quasi-aposymbionts), and most of them died. When they do not die, fitness parameters are compromised, as denoted by the delay in the developmental time, with the females showing important morphological and reproductive defects, not being able to develop normal oothecae and to reach the G3 as aposymbionts. Even though we have been able to develop quasi-aposymbionts, using this methodology, it is not currently feasible to obtain real aposymbionts, reinforcing the essentiality of *Blattabacterium* in the insect physiology, a role that cannot be played by the microbiota in its absence. Thus, the microbiota role must be another.

### 3.3. Effect of Blattabacterium Load on the Structure and Composition of the Gut Microbiota

**Figure 10:**
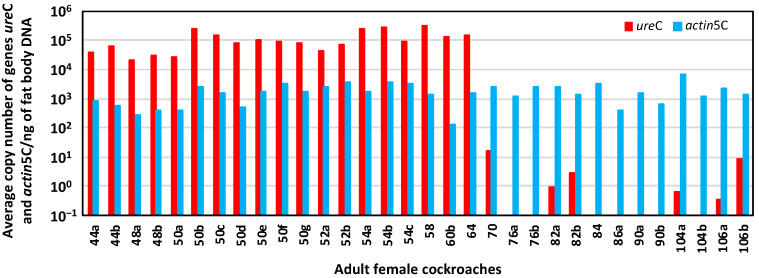
qPCR of fat body DNA of 32 adult females from the mixed population M. Log representation of the average copy number of genes *ure*C and *actin*5C per nanogram of fat body DNA for each female analysed. The number in the name of the sample indicates the day in which the adult ecdysis took place, counting from the start of the G2 mixed population M, and the letter identifies each of the individuals that passed to the adult stage the same day.

**Figure 11:**
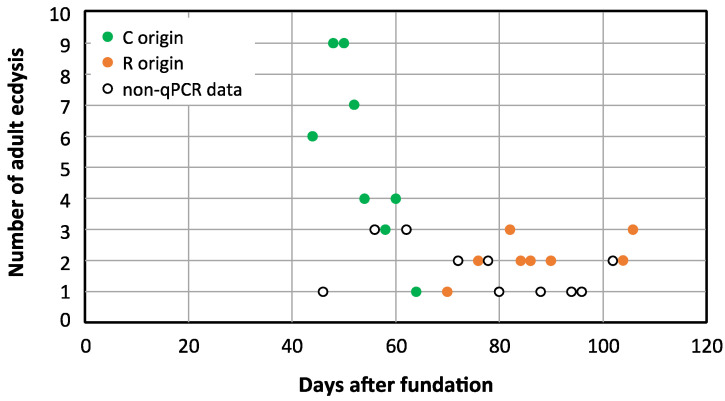
Distribution of adult ecdysis over time in the mixed population M. The graph displays the number of individuals (*y*-axis) that passed to the adult stage (males and females) after a specific day (*x*-axis), counting from the start of the G2 mixed population M. Green dots correspond to days 44 to 64, where qPCR showed that the females analysed came from the C population at G1, as they showed a normal load of *Blattabacterium.* Orange dots correspond to days 70 to 108 where analysed females came from the rifampicin-treated population at G1, based on their reduced load of *Blattabacterium*. Empty dots correspond to days where qPCR failed or only males passed to adult stage.

We demonstrated that rifampicin leads to a drastic decrease in the population size of *Blattabacterium* in G2, and that it was impossible to obtain at G3 an aposymbiotic population or with a more reduced load of endosymbiont. Therefore, to unravel the possible communication between the gut microbiota and the endosymbiont once one of them is altered, we decided to work with a stable quasi-aposymbiont population at G2 (Figure 1c). For this experiment, we started at G1 with two populations of synchronised sex-balanced adults, control (C) and treated with rifampicin 0.1 mg/mL during the adult stage and with faeces added from a control laboratory population (R). Once the antibiotic treatment ceased at 35 days, G2 was initiated with the nymphs hatched from both populations (C and R), to generate a unique mixed population (M) composed by 70 nymphs derived from the control population and 140 derived from the rifampicin-treated population (d0N at G2). Knowing that the neonatal digestive tract is free of microbes, faeces were supplied during the first ten days to promote a secure transmission of commensal microbiota [8,38]. In this way, we ensure the same external bacterial input to both types of nymphs in the mixed population, to avoid any environmental bias in the settlement of the gut bacterial communities. From the second experiment as well as from previous results, we know that cockroaches at G2 coming from the rifampicin-treated population must have a reduced *Blattabacterium* load (i.e., they are quasi-aposymbionts) compared with the ones coming from the control population [38,45]. Thus, in the M population, two sets of individuals with different *Blattabacterium* loads coexisted exactly in the same environment. The aim was to identify the individuals coming from each of the original population in the mix, based on the different *Blattabacterium* load, and to verify whether such difference affects, somehow, the composition and structure of the microbiota. As soon as an adult appeared in the mixed population (M), it was isolated from the rest and, after 10 days, each isolated female was dissected to obtain the fat body and the hindgut to investigate the endosymbiont load and the composition as well as, eventually, the functions of the gut microbiota. A total of 77 individuals reached the adult stage, the first one at 44 days and the last at 106 days of the nymphal stage, and 38 females were dissected. The estimation of the *Blattabacterium* load by qPCR is shown in Figure 10. Six qPCR reactions failed. The 19 successfully analysed samples corresponding to fat body DNA of females that appeared after 44 to 64 days of the nymphal stage oscillated between 10^6^ and 10^4^ copies of the *Blattabacterium ure*C gene per nanogram. However, the 13 analysed samples corresponding to females that appeared from the 70 to 106 days of the nymphal stage were below 10 copies per nanogram or even under the detectable threshold in the real-time qPCR assay. As expected, the copy number of the *B. germanica actin*5C gene per nanogram of fat body DNA was almost constant in all samples, regardless of the *Blattabacterium* load. These results allowed us to assign the individuals reaching adult stage to each original population, since previous qPCR studies revealed a difference of five orders of magnitude of the copy number of the *ure*C gene between control and G2 females coming from a population treated with rifampicin in G1 [38] (Figure 11), or even undetectable levels as in the second experiment. Considering that the individuals that needed 44–64 days to pass to the adult stage developed from the 70 G2 selected nymphs from the initial C population, 50 of them (26 males and 24 females, 19 of which were analysed by qPCR) reached the adult stage. On the other side, only 27 (13 males and 14 females, one of which failed in the qPCR assay) out of the 140 G2 selected nymphs coming from the rifampicin-treated population at G1 became adults after 70–106 days. These results confirm the negative effect of reducing the load of the endosymbiont in the insect development, extending the time from the nymphal to the adult stage, also causing an increase in mortality.

**Figure 12:**
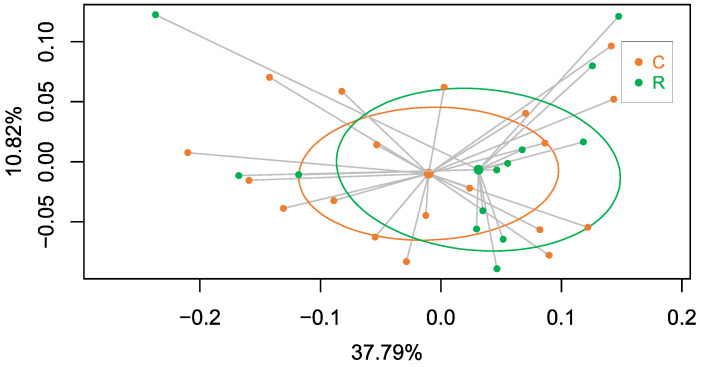
PCoA analysis based on Bray–Curtis distances after the beta diversity of the metagenomics data of the hindgut DNA samples from the mixed population M. The analysis corresponds to the same adult females that were analysed in Figure 10 and Figure 11 and to which the population of origin in G1 was assigned. The graph shows that females coming from the control population in G1 (green dots, C) and quasi-aposymbiont individuals coming from the rifampicin-treated population in G1 (orange dots, R) are located in the same region of the graph (Adonis test *p*-value = 0.66).

In addition, we conducted metagenomics analysis of all females in which the endosymbiont load had been determined to explore the putative structure of the corresponding hindgut microbiota. The number of reads from 32 female cockroaches after the quality check on raw sequence data coming from high throughput sequencing was 22,483,528 with an average number of 702,610.25 sequences per sample (maximum 1,188,500; minimum 301,319). The PCoA analysis (Figure 12) showed that the distribution pattern of the samples that came from the two G2 adult populations (C and R) was the same. The two clusters had a similar homogeneity level and the Adonis test validated the proof that the behaviour of the two gut microbiotas was not statistically significant (*p*-value = 0.66). This indicates that, even if the environmental conditions in G2 were the same for the nymphs of both populations and the faeces were supplied during the first 10 days of the nymphal stage to facilitate the colonisation of the hindgut, the composition and structure of the gut microbiota were not influenced by the change in the *Blattabacterium* load. Therefore, this result provided a negative answer to the questions posed in this work: “Is there a communication between the two symbiotic systems into *B. germanica*?” Further research needs to be conducted to empirically define the role of the gut microbiota, since it was shown that it was not able to compensate the lost roles of *Blattabacterium*.

## 4. Conclusions

The investigation carried out here aimed at better understanding if and how the two spatially isolated symbiotic systems of *B. germanica* interact, by analysing the changes associated with rifampicin treatment in both the *Blattabacterium* load and the gut microbiota composition.

In a first experiment, we learnt that rifampicin affects the sensitive bacteria of the gut microbiota early after treatment, with a delay of two days after faeces are supplied. Compared to the structure and abundance of the gut microbiota of the control population, the removal of the antibiotic allows for a recovery of most but not all the taxa to the expected level. Faeces seem to speed up the recovery, but other factors are also involved in it. In a second experiment, we tried to obtain aposymbionts by extending rifampicin treatment in one generation. We obtained a much-reduced load of *Blattabacterium* in the second generation of the few individuals that were alive and with a much-reduced fitness. Furthermore, these quasi-aposymbiotic individuals were not able to reach the third generation. In the third experiment a mixed population of quasi-aposymbiont and control individuals was formed and raised under the same environmental conditions, followed by microbiota analysis in the adults. The main conclusion is that the reduction in the *Blattabacterium* load did not influence the structure of the gut microbiota under the same environmental conditions.

Overall, our results indicate that the gut microbiota cannot replace the essential endosymbiont and that there is no interaction between the two symbiotic systems. Yet, additional functional investigations, including other studies with different classes of antibiotics and with germ-free individuals, need to be performed in order to clarify the specific functions properly and to understand the advantages that these two coexisting symbiotic relationships confer to the insect, so that a comprehensive view of the system can be reached. 

## Data Availability

The data for this study have been deposited in the European Nucleotide Archive (ENA) at EMBL-EBI under the study accession numbers PRJEB62131 and PRJEB62187.

## Supplementary Materials

The following supporting information can be downloaded at: https://www.mdpi.com/article/10.3390/biology12070955/s1, Figure S1: Heatmap and clustering based on taxon composition and Z-score transformed relative abundance of the gut microbiota corresponding to d2, d4 and d10 of treatment; Figure S2: Heatmap and clustering based on taxon composition and Z-score transformed relative abundance of the gut microbiota corresponding to 10, 40 and 50 days of recovery after treatment cessation (d20, d50 and d60 time points); Table S1: Relative abundance at family level for all samples from the treatment phase; Table S2: Relative abundance at family level for all samples from the recovery phase; Table S3: *p*-values of the Wilcoxon signed-rank test for paired combinations at treatment and recovery phases.
