# Supplementary material for: Exploring Gut Microbial Dynamics and Symbiotic Interaction in Blattella germanica Using Rifampicin
Source: Biology (Basel). 2023 Jul 3;12(7):955. doi: 10.3390/biology12070955 (PMC10376618; doi:10.3390/biology12070955)
Supplement: Supplementary file 1 [file biology-12-00955-s001.zip › Figure S1.pdf]

Exploring Gut Microbial Dynamics and Symbiotic Interaction in *Blattella germanica* Using Rifampicin

Monica Cazzaniga, Rebeca Domínguez-Santos, Jesús Marín-Miret, Rosario Gil, Amparo Latorre and Carlos García-Ferris

Figure S1

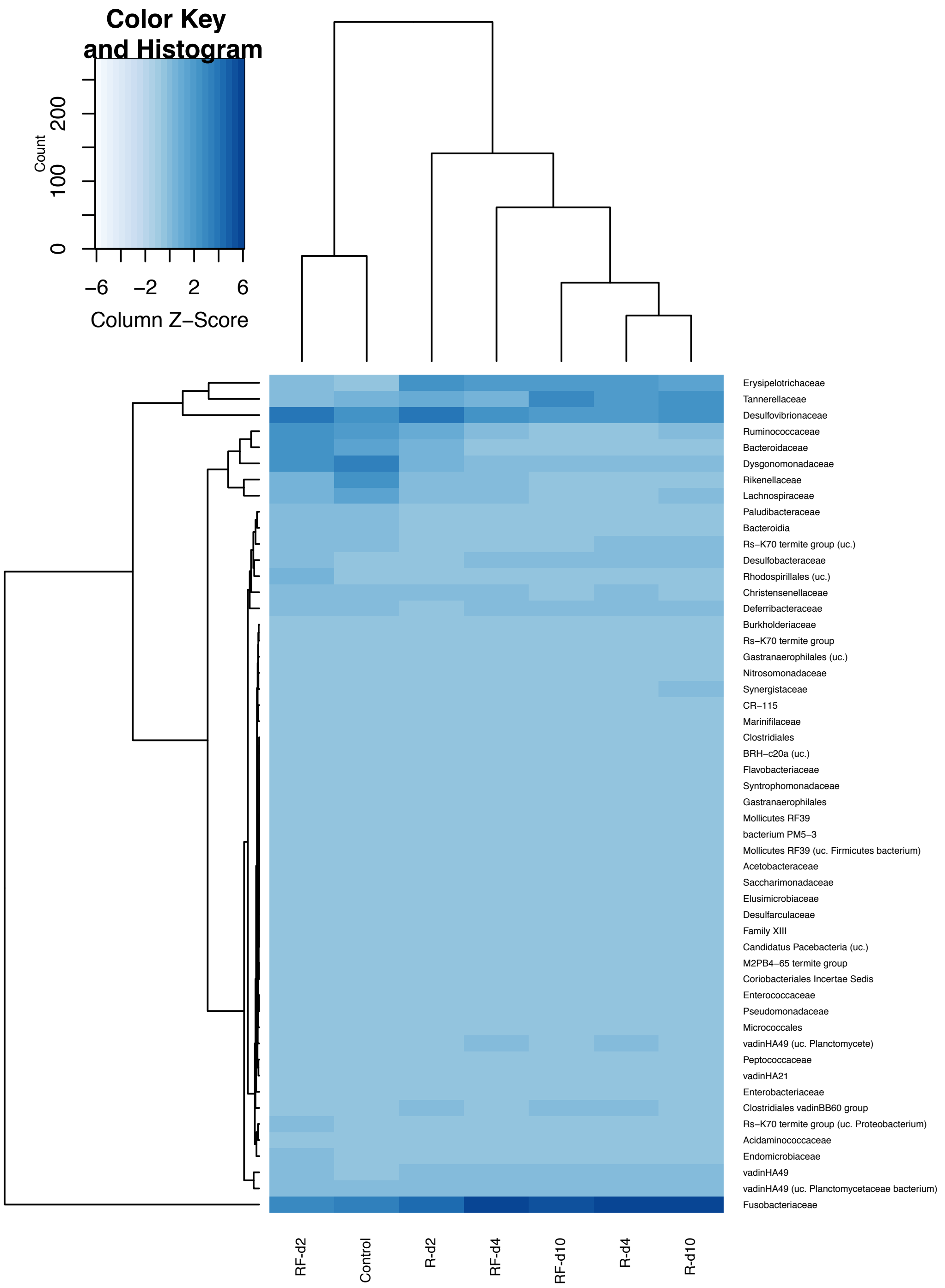

Figure S1. Heatmap and clustering based on taxon composition and Z-score transformed relative abundance of the gut microbiota corresponding to d2, d4 and d10 of treatment. Three populations were analysed: control (C, corresponding to grouped C-d2 to C-d10), treated with rifampicin (R), and treated with rifampicin and faeces (RF). Different experimental conditions are shown on the *x*−axis and the family of the taxa identified in each sample on the *y*−axis. The shade of colours was related to the Z-score of these taxa in each sample; light and dark blue indicate lower to higher abundance, respectively.
