# Supplementary material for: Exploring Gut Microbial Dynamics and Symbiotic Interaction in Blattella germanica Using Rifampicin
Source: Biology (Basel). 2023 Jul 3;12(7):955. doi: 10.3390/biology12070955 (PMC10376618; doi:10.3390/biology12070955)
Supplement: Supplementary file 1 [file biology-12-00955-s001.zip › Figure S2.pdf]

Exploring Gut Microbial Dynamics and Symbiotic Interaction in *Blattella germanica* Using Rifampicin

Monica Cazzaniga, Rebeca Domínguez-Santos, Jesús Marín-Miret, Rosario Gil, Amparo Latorre and Carlos García-Ferris

Figure S2

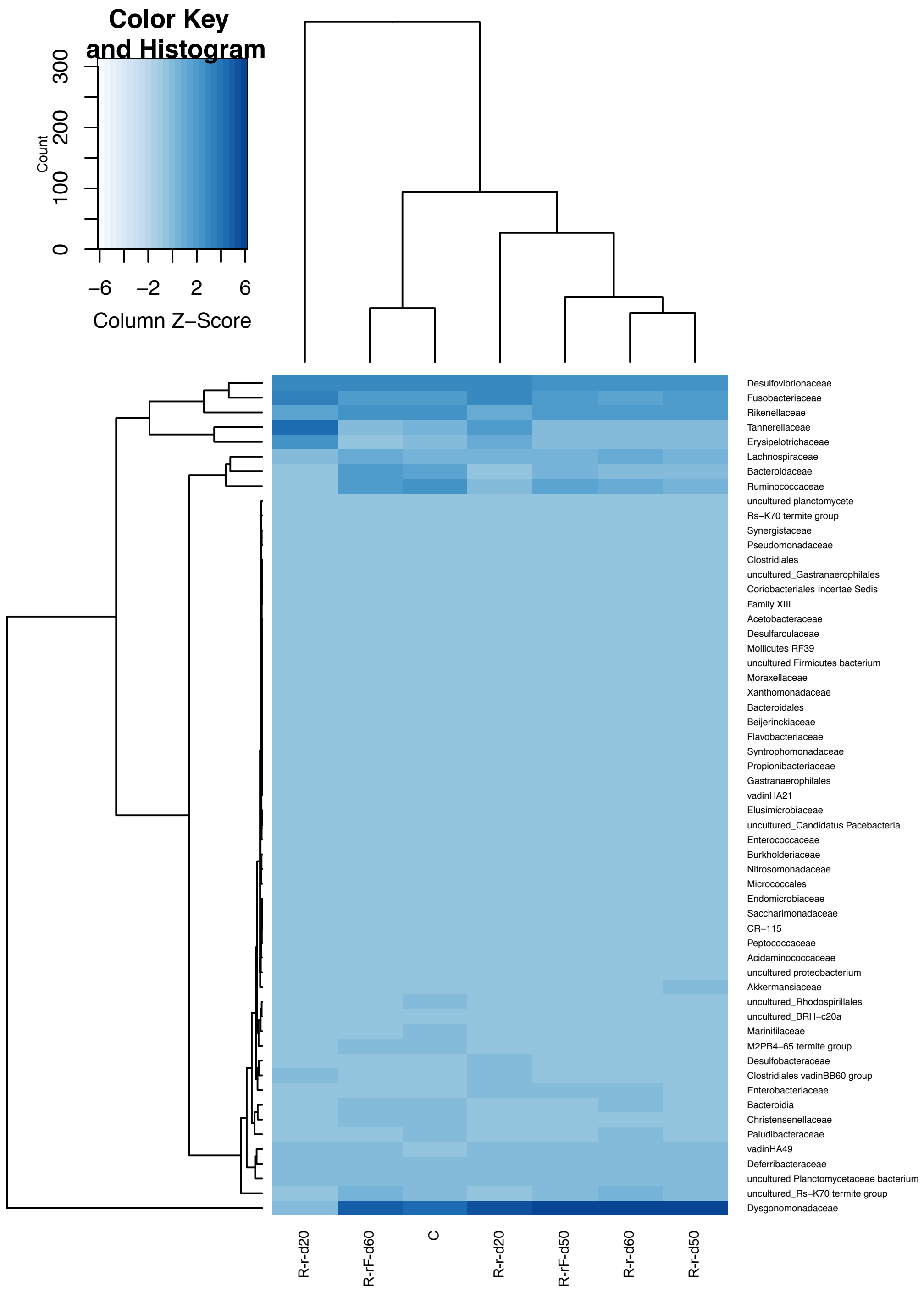

Figure S2. Heatmap and clustering based on taxon composition and Z-score transformed relative abundance of the gut microbiota corresponding to 10, 40 and 50 days of recovery after treatment cessation (d20, d50 and d60 time points). Three populations were analysed: control (C, corresponding to grouped C-d20 to C-d60 samples), rifampicin treated population recovered without faeces (R-r) and rifampicin treated population recovered with added faeces (R-rF). Different experimental conditions are shown on the *x*–axis and the family of the taxa identified in each sample on the *y*–axis. The shade of colours was related to the Z-score of these taxa in each sample; light and dark blue indicate lower to higher abundance, respectively.
